# ‘What is the most important lesson you learnt as a neuroscience master's student?’ A single-question study in postgraduate neuroscience education

**DOI:** 10.1186/s12909-024-05970-6

**Published:** 2024-10-29

**Authors:** Stefano Sandrone

**Affiliations:** Department of Brain Sciences, Faculty of Medicine, Imperial College London, London, UK

**Keywords:** Medical education, Neuroscience teaching, Neuroscience education, Attitudes, Identity, Skills, Community, Knowledge

## Abstract

**Introduction:**

Neuroscience is a young discipline and an expanding field of study and research. The number of neuroscience postgraduate courses has risen much more rapidly than in any other field of biomedical research. However, from a scholarship perspective, the master's experience has been understudied. This work focuses on the answers given by a group of neuroscience alumni, a rarely examined academic cohort, to a straightforward question: ‘What is the most important lesson you learnt as a master's student?’.

**Methods:**

Thematic analysis was performed on 27 entries hosted by a public-facing blog of a STEM-intense university across four academic years.

**Results:**

Four themes emerged: Skills, Theoretical knowledge, Attitudes and Community. Beyond replicating previous findings on the importance of skills and theoretical knowledge, an ‘imbalance’ between them has been reported for the first time. What emerges in our work is the overwhelming importance the alumni place in attitudes, especially resilience, and in the social/community aspects of learning, along with the role played by fellow students, faculty and laboratory colleagues.

**Discussion:**

The master's level can be the ideal one for increasing awareness of resilience and learning how to make the most of it. Institutions should consider strategies for strengthening informal learning and supporting the development of professional identities. These findings can be of interest to a wide range of neuroscience educators and provide useful insights for designing effective postgraduate training courses. Future works can investigate the *how* beyond the *what* and explore the roles played by attitudes, emotions and feelings across neuroscientists’ career stages.

## Introduction

The expression ‘postgraduate education’ identifies the learning period after the first graduation. It encompasses Master's courses, such as the Master of Science (MSc) and Master of Research (MRes), and the PhD training. About three decades ago, the postgraduate experience shifted from ‘being a fringe activity in higher education institutions to commanding a role that takes centre stage’ [11]. The beginning of the new millennium witnessed ‘proliferation of masters level programmes’ [14, 31, 54]. This was encouraged by governmental indications worldwide aimed at broadening international recruitment and increasing global mobility [34].

Master's courses are now a significant and structural part of lifelong learning, and the number of courses and students is still increasing [34, 45]. The modalities of teaching delivery and the diversity of these courses are more varied than ever [27]. Yet one shared highlight of master's courses in STEM is to provide a ‘direct linkage between teaching and learning activities and research’ [46, 52], which can potentially increase their employability for future career opportunities [28].

However, despite the prominence of the master’s experience, it has attracted less scholarly research interest than undergraduate and continuing education or doctoral studies [23] and ‘studies of higher education have focused on bachelor’s and doctoral education, with less emphasis on master’s students’ [27]. In parallel, a few reports exist on MSc/MRes courses in neuroscience [47]. Although neuroscience is a young field of study and research, it is rapidly changing the landscape of education and science. To give an idea of the magnitude of its expansion, it was reported in 2016 that the number of postgraduate courses in neuroscience has risen much more rapidly than in any other field of biomedical research [1]. The global neuroscience market for neuroscience ‘could grow to $721 billion by 2026’ [40]. It has also been forecasted that neuroscience will be at the nexus of several societal topics beyond medicine, from consumerism to the justice system [4, 42].

Considering the established prominence of the master's experience, the limited number of studies, the rising role of neuroscience and the importance of gaining insights to design effective postgraduate training courses, this study focuses on a rarely examined academic cohort, namely a group of neuroscience master's alumni, by asking them a simple and straightforward question: ‘What is the most important lesson you learnt as a master’s student?’.

## Methods

A secondary analysis of the entries of the *Alumni Brains* blog (http://wwwf.imperial.ac.uk/blog/alumni-brains/) was conducted. The blog is hosted on the website of a STEM-intense, top university in the United Kingdom and was designed and launched by the author in July 2020. It was conceived shortly after the start of the COVID-19 pandemic as a way to create a ‘virtual bridge' between different cohorts of students. It features individual interviews with alumni, namely former students who had completed either a full-time MSc or a full-time MRes in neuroscience at the same university across one of four academic years (2018/2019, 2019/2020, 2020/2021, 2021/2022). All the alumni enthusiastically accepted the invitation to be interviewed within a few days of receiving the invitation email. The curricula of the two courses are similar. While the MRes consists of three laboratory rotations across 12 months, the MSc has a 6-month teaching component and a 6-month laboratory project.

Participants were contacted by the author, then a Senior Teaching Fellow for both courses, via email on the days following the final exam. Those who agreed to be interviewed received the questions via email and answered them in writing, asynchronously. No limit to the number of words to be used was provided and no editing was done before publication (beyond a minimal grammar check or correction of spelling mistakes). The alumni were given about two weeks to answer the same questions in the same order, of which only one was analysed here; as the blog is public-facing, the participants waived anonymity. The overall set of questions included: What is your name? Where are you from? To which class do you belong? Where and what did you study before joining the master's course? How did you find your master's experience? Which research project did you work on? Where are you now? What are you working on? What is the most important lesson you learnt as a master's student? How did the master's programme help you get to where you are now?

Twenty-seven alumni (twenty-two females and five males) were interviewed (A1-A27). Their interviews were published online between July 2020 and April 2024. The twenty-seven answers to the question ‘What is the most important lesson you learnt as a master's student?’ were analysed in this study: the interview question acted as a prompt in the process, and the thematic analysis [13, 38] was conducted by the author, who is an experienced educator and scholar. Two steps within the coding process [41, 53] were followed: open coding and axial coding, which led to identifying first-order and second-order codes. The codes were not defined a priori but emerged from the thematic analysis [44]. 

## Results

Four themes were identified via the thematic analysis: Skills, Theoretical knowledge, Attitudes and Community.

### Skills

For many alumni, the most important lesson learnt revolved around learning new skills and being exposed to new experiences, which were mentioned both as a new opportunity (coding for A7, new project-related skills for A16, along with teamwork and collaboration) but also learning how to work independently to become a neuroscientist. Aligned with this, A18 said: ‘I realise how lucky I am to have gained this skill and how key it is to become a young researcher’. A18 also noted the scientific independence that such skills could lead up to. A20 further expanded on this aspect; for them, the most important lesson learnt was how ‘to solve a problem independently, to work independently as well as in a team’. This was echoed by A21, who linked this to the agenda-setting aspect of the life of a scientist, having learnt ‘how to be more independent and drive my own research’. Priority setting and time management were mentioned also by A17 (to ‘focus on the priorities’) and A25 (‘time management and study strategies’). A13 mentioned a keen eye for detail and forecasting what could go wrong to avoid it: ‘It forced me to pay attention to every detail of my experimental protocol and to try to understand what could go wrong – for example, understanding optimal conditions for your tissue and antibodies’. A15 focused on presentation skills, highlighting that the course ‘has taught me to speak up more and be brave in presenting my ideas for discussion’. A26 noted the importance of ‘personal branding and networking’ (Table 1).

**Table 1:**
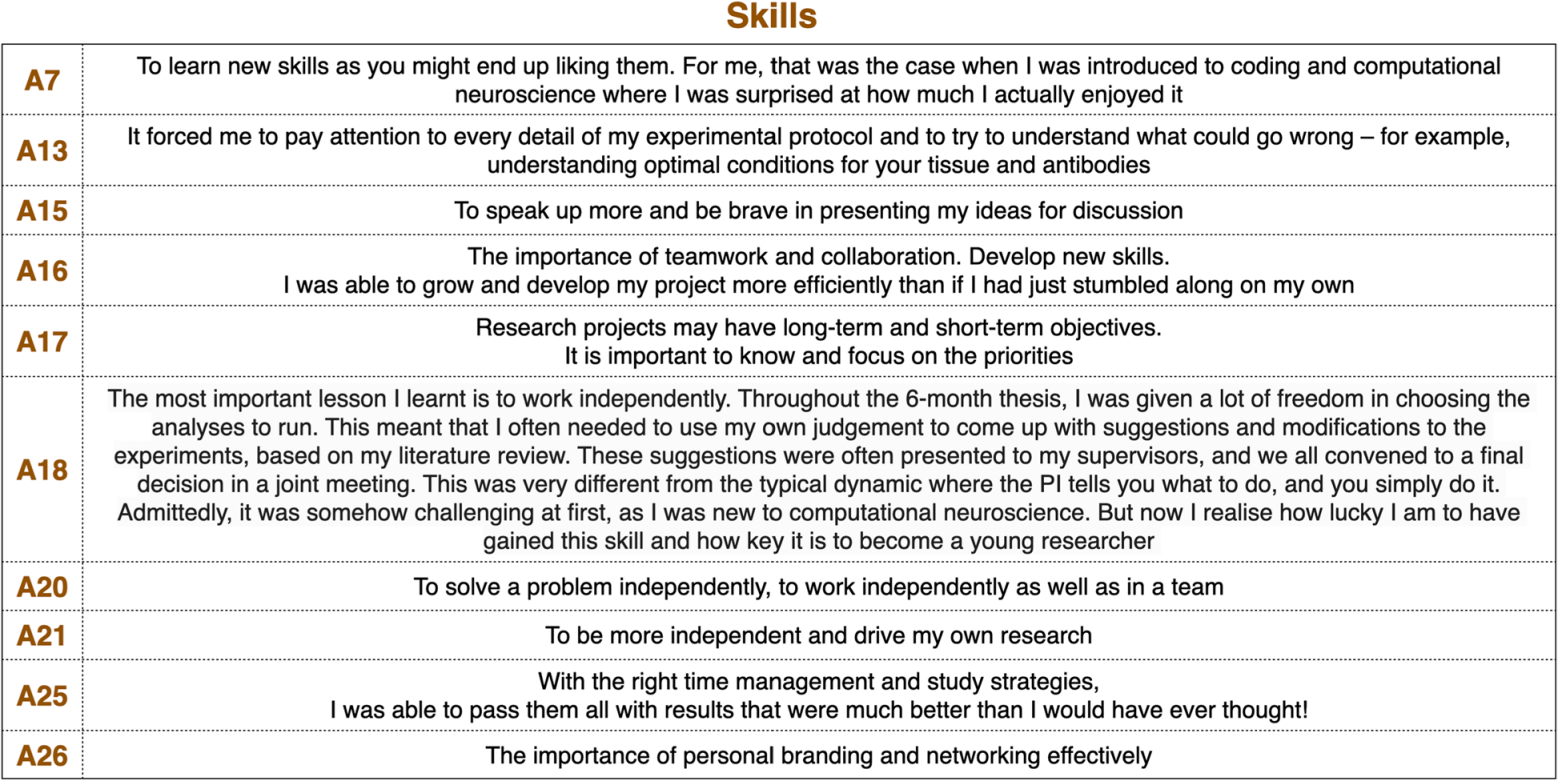
Skills: key answers

### Theoretical knowledge

A27 said that the most important lesson learnt was ‘the knowledge I acquired during the taught modules, which inspired me towards pursuing a career in academia’. This was reiterated by A3: ‘building on the knowledge (…) acquired during my undergraduate studies’ to ‘better integrate information from different disciplines and use my skills to target specific scientific questions’, but also ‘pass on our knowledge to produce remarkable work’ (in A16’s words). On a similar interdisciplinary note, the course helped some to realise that ‘scientific understanding is limited when appraised from the edges of a single discipline’ (A12) (Table 2).

**Table 2:**
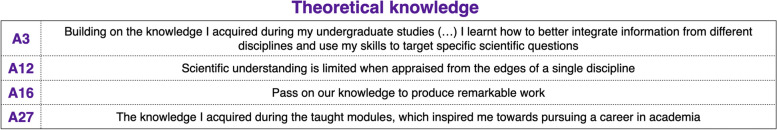
Theoretical knowledge: key answers

### Attitudes

Resilience was, by far, the most important lesson learnt during the course. This was reiterated from different perspectives by different alumni, who voiced many aspects of resilience, from never giving up (A7 and A1, who added: ‘Let the tough competition challenge your potential’), to maintaining ‘resilience and motivation’ (A22), ‘dedication and perseverance … my constant reminder was that no remarkable achievement is obtained without effort’ (A6), ‘Perseverance – work hard, have faith, never give up!’ (A14), ‘to persevere and to push myself’ (A9). A24 mentioned proactivity as a valuable lesson learned (alongside not waiting ‘for an opportunity to fall into your lap’), and both A2 (‘to always say “yes” to opportunities’) and A11 (‘to be open-minded to learning new things—and fast!’) echoed it. Instead, A8 and A25 focused on self-confidence (‘to have more confidence in my own abilities’ and ‘Refusing to believe that I cannot do something is the most important thing I learned from this course’, respectively), similar to A15 and A16. A27 reinforced the ‘can-do’ attitude: ‘I learnt that even when you are a master's student, you can make a significant contribution towards outstanding discoveries in your field’. A4 described the importance of leaving the comfort zone; in their own words, the importance of ‘getting comfortable with being uncomfortable. During my MSc, I learned how to be comfortable despite not knowing all the answers, tear my results apart to make sure they are rock-solid, and learn more every day’. Flexibility (in light of pandemic changes to teaching and learning) was also mentioned (by A19). A13 underlined that the course taught them ‘to be kinder to myself – or else I would have been a bundle of stress! I experienced for the first time a feeling that all scientists are accustomed to – the disappointment of when an experiment hasn’t worked’. A23 invited all to ‘Be true to science, do not ignore anything that seems not right, however tiny it might be’ (Table 3).

**Table 3:**
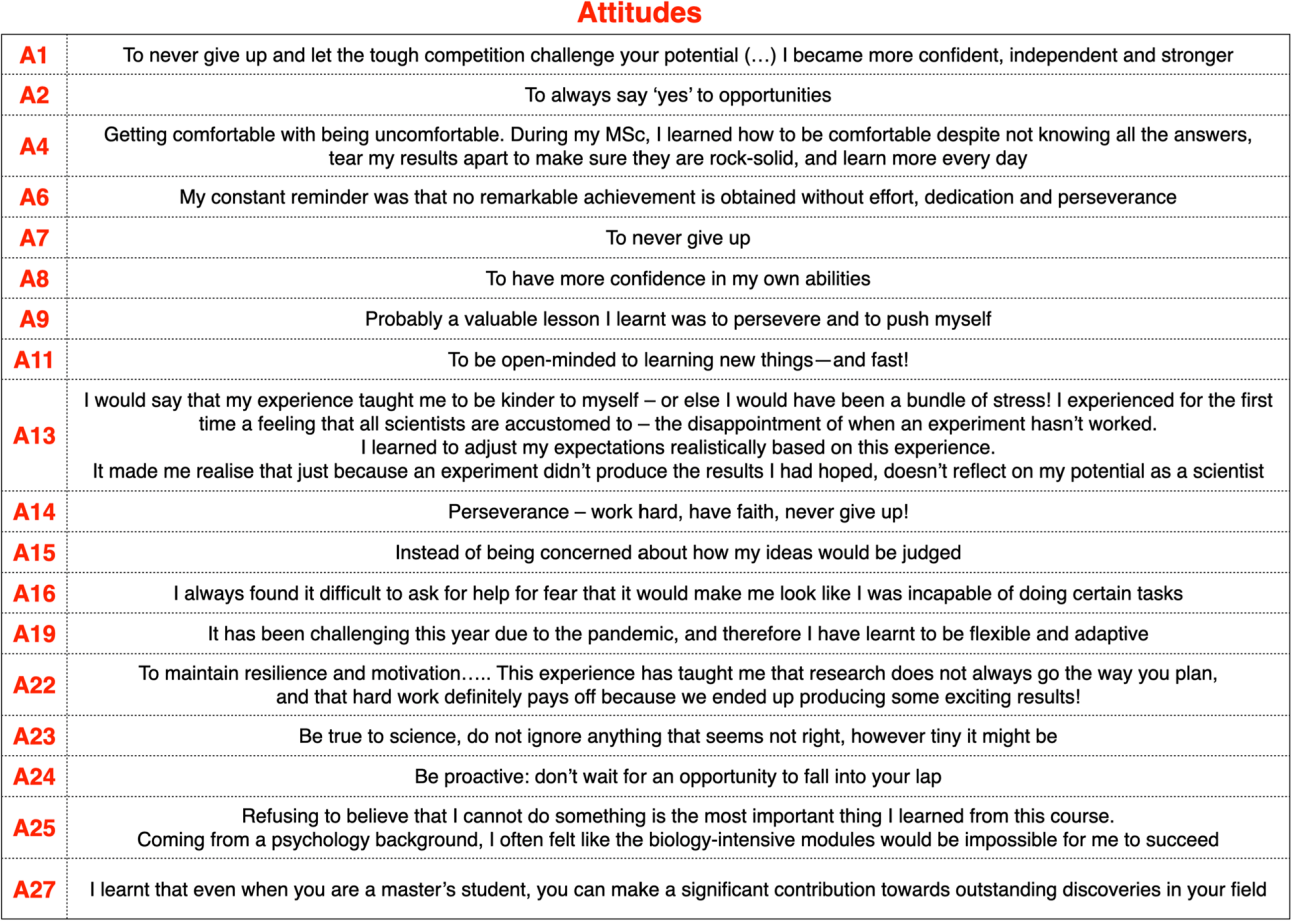
Attitudes: key answers

### Community

For several alumni, the people around them have been the most important highlight of the year. Fellow students, teachers, supervisors and colleagues in the laboratory were inspirational for their advice (A21), support (A8: ‘reminded me that they were once in my shoes’) and passion (A5: ‘For an aspiring researcher, it is important to be surrounded by people who share your passion. This will allow you to learn from them and become independent when progressing through your career’), but also for their ‘kind encouragement, inclusiveness and respect’ (A15). Peer learning was recognised to be an essential aspect (‘we were able to learn from each other’, A16), and this has been echoed by A21, who also emphasised how the social aspect of the teamwork contributes to defining a sensible experimental plan while ‘maintaining’ the ‘original idea’, thanks to ‘post-doc supervisors and the principal investigator’﻿. A10 stressed how fundamental it is to ‘look for mentors. They will be the most important thing in your career’. A24 summarised it: ‘Meet amazing new people and create lifelong friendships, network with excellent researchers (…), attend career and social events, explore all the opportunities available’. A16 was the only one who cited a bit of a barrier to asking for help: ‘Everyone in the course came with a different skillset … I was able to overcome those fears and ask for help from people around me who had the knowledge and experience’ (Table 4).

**Table 4:**
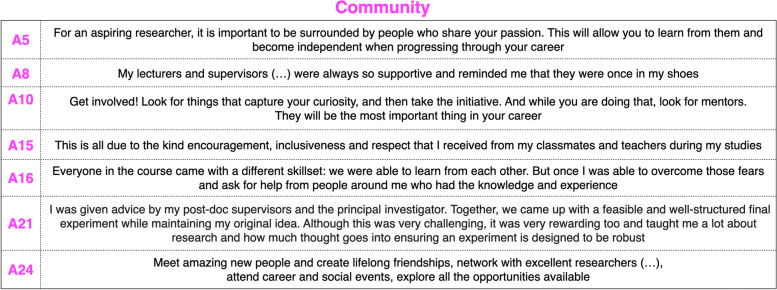
Community: key answers

## Discussion

Skills and theoretical knowledge were among the most cited lessons learnt. This aligns well with previously published works suggesting that conceptual and pragmatical skills are at the core of the master's experience [29], where ‘most of the teaching and learning in postgraduate education focuses on the transference of knowledge and procedural skills’ [50]. In this study, however, we took some steps forward. Not only did we replicate previous findings on the importance of skills and theoretical knowledge, which are discussed below, but we also reported an ‘imbalance’ between them, and we put attitudes and the role of the community surrounding neuroscience master's students under the spotlight.

The theoretical knowledge acquired was the most important aspect for a small number of alumni, whereas the skills acquired were given a prominent role by many. While there has been a recently reported growth in undergraduate neuroscience programmes [6, 20, 43], many of the students still join the master’s courses after completing undergraduate studies in different disciplines, from psychology to engineering, from medical studies to biotechnology, just to name a few [47]. Thus, specialism-wise, learning a skills-based ‘common language’ is crucial in the postgraduate context. The prominence of skills over theory taps into vocational learning and authenticity both from a learning perspective and assessment-wise [51] and is aligned with ‘practice-oriented concept as a principle of the professional training quality of the future specialists’ [16]. It might even reflect the increasing space given to active learning and ‘learning by doing’ in the neuroscience curriculum more generally [48, 49] and in the two courses specifically, where between 6 and 12 months are dedicated to the laboratory project, as it happens in many master's courses around the world.

The most cited skills were those leading to independence: working independently and in a team, priority setting, time management, ability to focus, presentation skills and networking. Such skills are highly valued by employers in STEM [3, 33, 57], and our findings agree with the importance of not losing ‘sight of training the soft and non-technical’ skills in postgraduate training [17, 50]. Yet while we successfully replicated some of the skills previously reported (such as communication and teamwork, explicitly found in the alumni’s answers), none of the alumni overtly mentioned critical thinking or emotional intelligence. However, what emerges in our work is the overwhelming importance placed by the alumni firstly in attitudes (18 out of the 27 alumni mentioned one or more aspects related to them, which makes this category the most cited one) and, secondly, in the social/community aspects of learning.

When analysing the attitudes, resilience was, by far, the most important lesson learnt, as voiced by several alumni. It is one of the predictors of academic flourishing along with grit, as shown by a study conducted with 101 postgraduate students, encompassing master's students and PhD students [5], which might overlap with the perseverance mentioned here and was indicated as a strong predictor among medical students in this and other publications [21, 35]. Persistence has been studied as a mediator between motivation and performance among students in medical education [19]. Yet postgraduate researchers have significantly lower well-being and resilience levels than the general population [12, 36]. It is detrimental to see this sudden change in a short time, namely between the master's and the PhD or the early postdoc, and ensuring that training programmes increase the students’ experience should be a priority. We, therefore, recommend that master's courses become the educational space to raise awareness on how to enhance and maintain resilience before young neuroscientists approach the following career stages.

Self-confidence, motivation, and proactivity have additionally been recognised as important lessons. Considering that ‘the application of knowledge requires skills and application of skills require confidence…designing, developing, focussing, and exposing students to…confidence inducers’ [2] might be an avenue to follow. Strategies for enhancing informal learning and supporting the development of professional identities are instrumental to transforming a group of learners with a shared set of values and ambitions in a ‘community of practice’ [55, 56], and we recommend they become part of the neuroscience postgraduate training at the master's level.

On a community-related note, alumni repeatedly mentioned fellow students, teachers and laboratory colleagues as key players. However, this enthusiasm contrasts with reports stating that being a scientist and an academic is often associated with a sense of loneliness [24, 37, 39]. In the postgraduate context, loneliness has been reported by students, who try to mitigate it by interacting socially, as revealed by a netnographic work with PhD students [25] and replicated in the following years with other experimental approaches [18]. In parallel with the resilience seemingly running low between the master’s and the postdoc stage, we need to counteract this drift. Considering the importance placed by master's students on the people around them and that a laboratory culture manifests itself in interpersonal relationships [22], facilitating social interaction can be a strategy to adopt to increase the students' experience [15, 25]. The master's level might be the ideal one to shape the research culture: improving the research environment [30] at the project stage should become a focus across institutions.

This work has limitations and strengths. Although independent prior coding by two researchers is usually recommended, this is a single-authored, single-centred, retrospective study, and the author is an experienced scholar. On the one side, adopting the one-question approach prevented the possibility of asking follow-up questions to clarify ambiguous aspects; however, on the other side, the answers to a single question might be easier to interpret than a multi-question survey [9, 26, 32]. The answers provided by the alumni consistently appeared straightforward, which was encouraging. Additionally, the interviewees were given no word limits to craft their answers, so they could freely explore (or choose not to explore) the topics as they wished and to the extent they wanted: embracing a notable amount of freedom was guaranteed. Although we do not have detailed demographic information about the interviewed sample, the sample size of this educational study is adequate to achieve data saturation and an in-depth description of the identified themes, which is a positive sign. This analysis covers four academic years, which is another strength. As the interviews were published online at an irregular rate (i.e., every n number of weeks), some of the alumni might have read their colleagues' answers before providing their own, but the fact that no two answers were identical is reassuring. Another strength is the systematic approach of asking the same questions in the same order to all the alumni, a standardisation that signals a rigorous methodological approach [10].

Did the alumni present themselves as ideal students [58] and wannabe-independent young scientists? Did they cite the people around them because they knew their answers would be publicly posted? Do the lessons learnt reflect only a *local* teaching environment or can we generalise these findings to all the master's courses in neuroscience globally, as they share certain features? The alumni knew their answers would have been posted on the university-branded blog, which might have led to a positive bias or social desirability [7, 8], and these questions can be the basis for future studies. Future educational works can use these findings as a starting platform to see if these results can be replicated in other academic contexts. A research avenue can be to ask the same question not only *immediately* after the end of the neuroscience master’s course (as in this work, where the alumni were interviewed just days after completion of their studies) but years after they finish their course to see if the lessons learnt in such a *delayed* framework differ. It would be interesting to plot the *immediate* and *delayed* lessons learnt against the alumni’s careers to understand if and how these played a role in shaping career choices. Future works can investigate the processes behind the themes that emerged here, i.e., via focus groups, to shed light on the *how* beyond the *what.* Additionally, how these important lessons can contribute to the sense of identity and the process of becoming a neuroscientist can be studied. Suggestions for future works include in-person interviews following a specific methodological framework for semi-structured interview design. Given the importance that the alumni placed on the attitudes, future studies can also examine the roles played by attitudes, emotions and feelings across neuroscientists’ career stages.

In conclusion, this work focused on a rarely examined academic cohort and explored the most important lessons learned during the master's experience across different academic years. The findings presented and discussed can be of interest to a wide range of neuroscience educators and provide useful insights for designing effective postgraduate training courses.

## Data Availability

The datasets analysed in this study are available online: https://blogs.imperial.ac.uk/alumni-brains/
